# Case-controlled study of tuberculosis in in-vitro fertilisation-embryo transfer and natural pregnancy

**DOI:** 10.1186/s12884-024-06260-1

**Published:** 2024-01-23

**Authors:** Jia-Lu Wei, Le Zhang, Yan-Ling Xu, Wei Gan, Min Qi, Xu-Wen Fu, Xiang Li

**Affiliations:** 1 Department of Radiology, The Third People’s Hospital of Kunming/Yunnan Clinical Medical Center for Infectious Diseases, No. 319 of Wujing Street, Guandu District, Kunming, 650041 China; 2 Department of ICU, The Third People’s Hospital of Kunming/Yunnan Clinical Medical Center for Infectious Diseases, Kunming, 650041 China

**Keywords:** In-vitro fertilisation and embryo transfer, Natural conception, Pregnancy, Tuberculosis

## Abstract

**Objective:**

To improve the understanding of the clinical features and imaging characteristics of pregnant women with and without in-vitro fertilisation-embryo transfer combined with pulmonary tuberculosis (TB).

**Methods:**

A retrospective analysis was conducted involving 50 patients with pregnancy who had pulmonary TB and were admitted to the Third People’s Hospital of Kunming (China) between 1 January 2017 and 31 December 2021. These patients were divided into an in-vitro fertilisation and embryo transfer (IVF-ET) conception group and a natural conception group according to the conception method. The clinical and imaging data were then collected and compared.

**Results:**

The mean age of the IVF-ET group (*n* = 13, 31.85 ± 5.84 years) was higher than in the natural conception group (*n* = 37, 27.05 ± 5.5 years). The proportions of fever, haematogenous TB and extrapulmonary TB in the IVF-ET group (92.31%, 84.62% and 76.92%, respectively) were higher than those in the natural conception group (40.54%,16.22%,27.03%,respectively). The percentage of patients with pregnancy who had intracranial TB (76.9%) in the IVF-ET group was higher than in the natural conception group (10.8%). The percentage of pregnancy terminations in the IVF-ET conception group (84.62%) was higher than in the natural conception group (48.65%). All the above results had statistically significant differences (*p <* 0.05).

**Conclusion:**

Overall, IVF-ET conception combined with extensive pulmonary TB lesions lead to heavy systemic toxic symptoms, severe disease and poor pregnancy outcomes. Therefore, screening for TB prior to performing IVF-ET is recommended.

**Supplementary Information:**

The online version contains supplementary material available at 10.1186/s12884-024-06260-1.

## Introduction

With the development of reproductive technology, in-vitro fertilisation and ‘test-tube babies’ have gradually become an option. However, new methods also create more challenges; for example, the potential side effects of pregnancy increase, and diseases can have a greater effect on pregnancy outcomes.

Tuberculosis (TB), caused by a *Mycobacterium tuberculosis* infection of the human body, can occur in all organs and tissue types of the body. The disease is a serious public health problem worldwide, with close to 10 million new TB cases per year and approximately 1.3 million deaths [1]. The disease is also one of the major non-obstetric aetiologies of maternal death [2]. Approximately 700,000 women reportedly die from TB each year, approximately 30% of whom are women of reproductive age between 25 and 44 years [3]. ‘Pregnancy with TB’ refers to a woman who is infected with TB during pregnancy or who experiences a pregnancy without recovering from the disease. Changes in the maternal immune function during pregnancy may increase the risk of developing TB [4]. The incidence of pregnancy with active TB in China is approximately 70 per 100,000, which is much higher than the 7.1 per 100,000 recorded in developed countries [5]. 

In clinical work, TB is often misdiagnosed in women with pregnancy as an upper respiratory tract infection due to limitations in examining the patient caused by their specific physical condition, thus delaying the opportunity for treatment [6]. Therefore, the early diagnosis and treatment of pregnancy complicated with pulmonary TB is vital. The complexity of the patient’s condition during pregnancy makes it even more challenging to treat them after they develop TB and can cause a series of adverse reactions, such as foetal abortion, deformity and death. With the development of in-vitro fertilisation and embryo transfer (IVF-ET), more infertile patients are choosing this method of conception [7]. Whether IVF-ET increases the chance of developing active TB in pregnancy, as well as its impact on pregnancy outcomes, has attracted wide clinical attention; however, no published reports currently exist on this topic. Since there is a high incidence of TB in Yunnan Province, and because pregnancy complicated with pulmonary TB is common in clinical work, the current authors’ aim was to conduct a case-controlled study of pulmonary TB in patients with pregnancy who have conceived naturally and via IVF-ET in a bid to enhance awareness of the disease and improve early diagnosis and treatment.

## Data and methods

### Study participants

Patients with confirmed pregnancy complicated with pulmonary TB in the Third People’s Hospital of Kunming (China) between 1 January 2017 and 31 December 2021 were selected for retrospective analysis. The patients were divided into two groups: an IVF-ET conception group and a natural conception group. All patients were briefed on the aims of the study and signed an informed consent form.

### Inclusion and exclusion criteria

#### Inclusion criteria

A diagnosis of pregnancy with TB was made with reference to the Chinese Health Industry Standard – Tuberculosis Classification (WS 196–2017) and the Chinese Health Industry Standard – Tuberculosis Diagnosis (WS 288–2017), with consistent TB imaging as referenced in the Imaging Diagnostic Criteria for Pulmonary Tuberculosis [6]. The presence of TB in pregnant women was further confirmed using an acid-fast bacilli smear, *M. tuberculosis* culture and *M. tuberculosis* Xpert assay. This study was approved by the hospital’s research ethics committee (batch no. 2,020,080,403), and informed consent was obtained from all the patients.

### Exclusion criteria

The following exclusion criteria were applied: (1) ectopic pregnancy; (2) patients without complete clinical data due to a hospital referral for treatment; and (3) patients who did not undergo an imaging examination by the hospital.

### Clinical data collection

#### Medical history and clinical manifestations

The relevant data were collected from the hospital’s electronic case system, which included patient history, clinical symptoms and signs, laboratory examination and prognosis.

### Tuberculosis aetiology

Patient sputum and bronchoalveolar lavage were collected for an acid-fast bacilli smear, *M. tuberculosis* culture and *M. tuberculosis* Xpert assay. A TB diagnosis was given when one of these tests was positive for *M. tuberculosis.*

#### Imaging examination

Computed tomography (CT) was performed using a GE Medical Systems LightSpeed VCT or BrightSpeed. The scanning tube voltage was 120 Kv, the tube current was selected using the auto-milliampere technique, the scanning layer thickness was 5 mm and the reconstruction layer thickness was 0.625–1.25 mm. Magnetic resonance imaging (MRI) was performed with uMR 588 or uMR 780. Prior to the CT and MRI examinations, the patients were informed of the possible adverse effects of the X-ray, iodine contrast, gadolinium contrast and MRI electromagnetic radiation on the foetus, and an informed consent form was signed. All patients underwent chest CT examination. During the process of chest CT examination, lead clothing was used to cover the non-scanned site. All patients underwent MRI of the brain and symptomatic organs such as the vertebral body and abdomen, as well as scanning for lymphadenopathy. In the IVF-ET group, 12 patients (12/13, 92.31%) underwent abdominal and vertebral MRI scans and 13 patients (13/13, 100%) underwent whole-body superficial lymph node ultrasonography; in the natural conception group, 18 patients (18/37, 48.65%) underwent abdominal and vertebral MRI scans and 37 patients (37/37, 100%) underwent whole-body superficial lymph node ultrasonography.

### Imaging analysis

The CT and MRI images were read blindly by two doctors with intermediate or above professional titles who had been engaged in TB imaging diagnoses for many years. If there was a disagreement between the two physicians, a third experienced doctor reviewed and confirmed the diagnosis. The following CT and MRI diagnoses were included in this study:

(1) Pulmonary TB image classification [8]: This was divided into primary pulmonary TB, haematogenous disseminated pulmonary TB and secondary pulmonary TB. Haematogenous pulmonary TB was further divided into acute haematogenous disseminated pulmonary TB, subacute haematogenous disseminated pulmonary TB and chronic haematogenous disseminated pulmonary TB.

(2) Imaging diagnosis of extrapulmonary TB: (a) intracranial TB, presented as meningeal TB or brain parenchyma TB. The imaging manifestations were meninges thickening, multiple TB nodules or tuberculoma of the brain parenchyma [9]; (b) thoracic TB, where the imaging manifestations were irregular, spindle or circular-like reinforced soft tissue masses, which could be accompanied by adjacent bone and cartilage destruction [10]; (c) tuberculous pericarditis, presented with pericardial thickening and a low-density shadow in the pericardial cavity [11]; (d) lymph node TB, where the imaging manifestations were ring-reinforced multiple lymph node enlargement with internal calcification [12]; (e) liver TB, with this diagnosis divided into the capsule and parenchymal types, which manifested on imaging as continuous or progressive enhancement of the capsule lesions or intraparenchymal liver lesions [13]; (f) spinal TB, mainly manifested as vertebral bone and intervertebral disc destruction or paravertebral abscess formation, which could be accompanied by post-process deformity [12]; and (g) pelvic TB, where the imaging manifestations were an irregular pelvic soft tissue mass with uneven density and uneven reinforcement on the enhanced scans [14]. 

The patients were treated with an initial anti-TB regimen of isoniazid, rifampicin, ethambutol and pyrazinamide. Anti-inflammatory treatment with glucocorticoids was additionally given to patients with complicated intracranial TB. If intrauterine foetal death, intrauterine infection or inevitable abortion occurred, or if any serious complications or concerns for the pregnant woman arose over the impact of diagnosis and treatment on the foetus, the pregnancy was artificially terminated.

### Statistical methods

Data analysis was performed using SPSS version 26.0 software. The measurement data were first checked for compliance with the normal distribution. If the data were normally distributed, the mean ± standard deviation (± s) was used. An independent samples t-test was used to compare the mean difference between the two independent samples. The count data were expressed using frequency (percentage), and the differences were compared using Fisher’s exact test. If the data did not follow a normal distribution, the Mann–Whitney U test was used to compare the non-parametric model. A *p-*value of < 0.05 was considered statistically significant.

## Results

### Clinical data

**Fig. 1:**
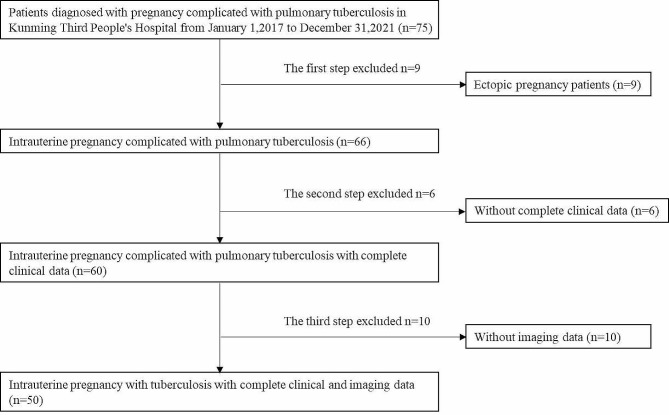
Roadmap of the inclusion and exclusion criteria for the study

A total of 75 patients diagnosed with pregnancy with TB in the Third People’s Hospital of Kunming between 1 January 2017 and 31 December 2021 were reviewed, none of whom were screened for TB before pregnancy. Based on the exclusion criteria, 9 (12%) patients with ectopic pregnancies, 6 (8%) with incomplete clinical data due to hospital referral and 10 without an imaging examination at the hospital were excluded from the study. A total of 50 (66.7%) cases of pregnancy with TB were finally included, and their clinical and imaging data were collected. Figure 1 shows the roadmap for the inclusion and exclusion criteria.

Based on the inclusion and exclusion criteria, the 50 included patients with pregnancy complicated with pulmonary TB were divided into the IVF-ET (*n* = 13; 26%) and natural (*n* = 37; 74%) conception groups. The patients in the IVF-ET conception group were aged 23–43 years, with a mean age of 31.85 ± 5.84 years. The patients in the natural conception group were aged 16–43 years old, with a mean age of 27.05 ± 5.5 years old. The mean age of the patients in the IVF-ET conception group was greater than that of those in the natural conception group (*p* = 0.017), and the difference was statistically significant. The patients in the IVF-ET conception group were at 13–26 weeks of gestation, and the mean gestational week was 19.15 ± 4.24. The patients in the natural conception group were at 6–40 weeks of gestation, and the mean gestational week was 21.18 ± 11.18. There was no significant difference in gestational week between the two groups at the time of diagnosis for pulmonary TB (*p =* 0.544) (Table 1).

**Table 1:** Clinical symptoms, laboratory examinations, imaging findings, and outcomes in both groups

	IVF-ET conception group (*n*=13)	Natural conception group (*n*=37)	P
Clinical symptoms			
Fever	12(92.31)	15(40.54)	0.001
Cough and expectoration	5(38.46)	22(59.46)	0.19
Hemoptysis	0(0)	6(16.22)	0.293
Pectoralgia	0(0)	6(16.22)	0.293
Dyspnea	2(15.38)	4(10.81)	0.66
Positive tuberculosis etiology	4(30.77)	16(43.24)	0.143
Combined with hematogenous disseminated tuberculosis	12(92.31)	6(16.22)	0.000
Combined with extrapulmonary tuberculosis	10(76.92)	10(27.03)	0.002
Termination of pregnancy	11(84.62)	18(48.65)	0.024
Gestational age for screening (week)	19.15 ± 4.24	21.18 ± 11.18	0.544
Gestational age for termination (week)	14.89 ± 8.12	19.6 ± 4.55	0.104

Note: IVF-ET in-vitro fertilisation and embryo transfer

### Clinical symptoms

In the IVF-ET conception group, 12 patients (92.31%) developed fever, 5 (38.46%) had a cough and expectoration and 2 (15.38%) developed dyspnoea. There were no symptoms of haemoptysis or chest pain. In the natural conception group, 15 patients (40.54%) developed fever, 22 (59.46%) had a cough and expectoration, 6 (16.22%) had haemoptysis, 6 (16.22%) had chest pain and 4 (10.81%) had dyspnoea. The proportion of fever was higher in the IVF-ET conception than in the natural conception (*p =* 0.001) group. The two groups had no significant difference in terms of the other clinical symptoms (Table 1).

### Pathology of Tuberculosis

Four patients (30.77%) in the IVF-ET conception group tested positive for *M. tuberculosis*, and 16 patients (43.24%) in the natural conception group tested positive for this bacterium, with no significant difference between the two groups (Table 1).

### Imaging performance

In terms of haematogenous disseminated TB, there were 12 (92.31%) cases in the IVF-ET and 6 (16.22%) in the natural conception group. The IVF-ET group exhibited a higher proportion of haematogenous disseminated TB than the natural conception group, with a significant difference between the two groups (*p =* 0.000). Ten patients in the IVF-ET group (76.92%) had the complication of extrapulmonary TB, and 10 patients in the natural conception group (27.03%) had this complication. The proportion of extrapulmonary TB was higher in the IVF-ET than in the natural conception group (*p =* 0.002), and the difference was statistically significant (Table 1).

Among the 50 patients studied in this group, 20 had extrapulmonary TB, including 14 with intracranial TB, among which 10 cases (76.9%) were in the IVF-ET conception group and 4 (10.8%) were in the natural conception group. The proportion of intracranial TB was higher in the IVF-ET conception than in the natural conception group (*p =* 0.000), and the difference was statistically significant. There was 1 case with a combination of chest wall TB, pelvic cavity TB, liver TB, lumbar spine TB, lymph node TB and tuberculous pericarditis.

**Fig. 2:**
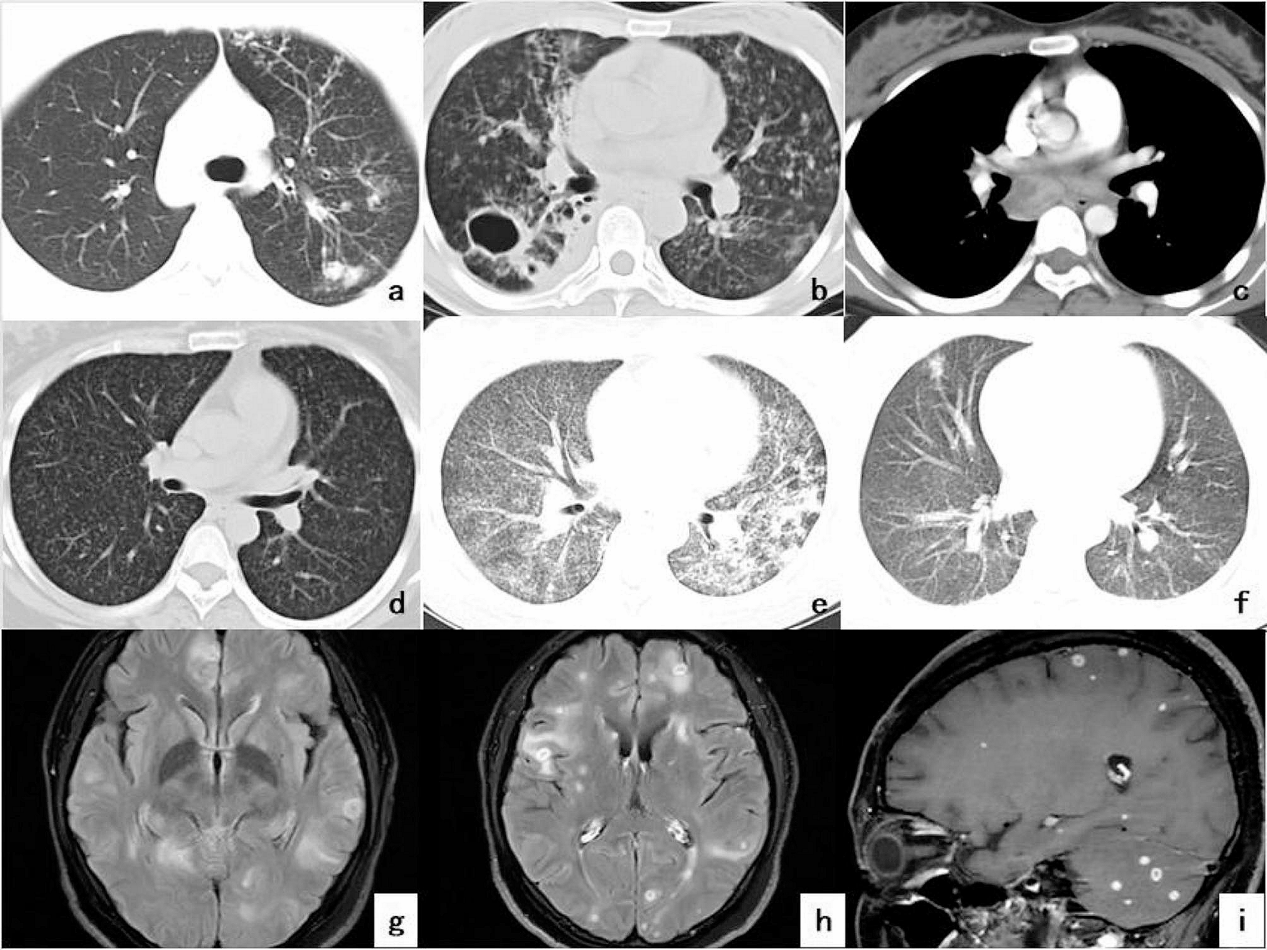
Imaging findings of pregnancy complicated with pulmonary tuberculosis and intracranial tuberculosis. **a**. shows the nodular and patchy shadows of the left lung, with local Tree-in-Bud sign; **b**. The cavity and multiple patchy consolidation in the right lung; **c**. Enlargement of intrathoracic lymph nodes, and the ring enhancement of enhancement scan; **d**-**f**. Three different patients of acute hematogenous disseminated tuberculosis, presenting a “three uniform” millet nodule shadow, partial patchy fusion; **g**-**i**. MRI findings in the same patient; **g**. the T2Flair transverse position. Multiple intracranial nodules with slightly higher T2 signal and partial peripheral brain parenchyma edema; **h**. For transverse images of T2Flair enhancement. Focal nodular and annular enhancement; **i**. Sagittal images T1WI enhancement. Focal nodular and annular enhancement

There were 32 cases of pregnancy with secondary TB, including 29 cases (90.62%) with nodule shadow and consolidation (Fig. 2a), 12 cases (37.50%) with pulmonary cavity formation (Fig. 2b), 12 cases (37.50%) with the tree-in-bud sign, 12 cases (37.50%) with pleural effusion, 5 cases (15.62%) with lymphadenopathy (Fig. 2c) and 11 cases (34.38%) with bronchiectasis. A total of 18 cases of pregnancy complicated with haematogenous disseminated TB were acute and manifested with miliary nodules of uniform size, distribution and density, as well as partial fusion (Fig. 2d–f). All the patients with intracranial TB presented with multiple nodules in the meninges and brain parenchyma, oedema of the surrounding brain parenchyma and nodular and annular enhancements upon enhanced scanning (Fig. 2g–i). The chest wall TB mainly exhibited spindle swelling of the chest wall soft tissue and focal ring enhancement on the enhanced CT. The patients with pelvic TB exhibited a quasi-circular low-density shadow and abdominal effusion, with ring enhancement on enhanced scanning, whereas those with liver TB exhibited a slightly lower density quasi-circular shadow of the liver sub-capsule, ring enhancement on enhanced scanning, congestion change of the adjacent liver parenchyma and obvious enhancement of the liver capsule and adjacent peritoneum. Finally, lumbar TB exhibited bone destruction of the lumbar 2–4 vertebrae, intervertebral disc destruction and bilateral psoas abscess formation, whereas lymph node TB exhibited multiple cervical lymph node enlargements with ring enhancement and TB pericarditis exhibited marked thickening of the pericardium and pericardial effusion.

### Pregnancy outcomes

Two patients in the IVF-ET conception group (15.38%) continued their pregnancy until delivery. One baby was delivered in another hospital following discharge at 27 weeks of gestation. The other patient had a natural labour at 28 weeks, but the child eventually deteriorated, and the treatment was abandoned. The remaining 11 patients (84.62%) terminated their pregnancy (11 cases were terminated after 28 weeks). The reason was that the pregnant women had TB that was too severe, and the doctor advised termination of the pregnancy. In the natural conception group, 19 patients (51.35%) continued their pregnancy, 9 of whom were discharged from hospital. Meanwhile, 10 patients had a natural birth (1 patient at 29 weeks, 3 at 36 weeks and 6 at full term), with the remaining 18 patients (48.65%) terminating their pregnancy. Among them, 11 patients underwent an induced abortion at less than 12 weeks of gestation due to concerns over TB and the influence of drugs on the foetus. In addition, 6 patients underwent an induced abortion at 12–28 weeks of gestation due to TB aggravation, and 1 case underwent induced intrauterine labour after 28 weeks of gestation due to TB aggravation. All patients were given conventional anti-TB treatment and related adjuvant therapy following delivery or termination of the pregnancy (Table 1).

## Discussion

^The clinical manifestations of^ TB ^mainly in^volve ^the systemic^ presentation of ^fever^ as well as ^fatigue, weight loss and respiratory symptoms^, ^such as a cough, expectoration, haemoptysis, chest pain and dyspnoea. A^ll ^of the 50 patients in this study had systemic poisoning^ and ^respiratory symptoms^. The first diagnosis of pregnancy with TB is generally at 18–21 weeks of gestation, which is related to the physiological reactions during pregnancy, such as drowsiness, fatigue and poor appetite. These are similar to the early systemic symptoms of TB, thus making the diagnosis of pregnancy with TB difficult.

An IVF-ET pregnancy combined with TB makes clinical diagnosis and treatment more complicated, and female genital TB (FGTB) is an important cause of female infertility [15]. However, since the onset site of FGTB is hidden and the manifestation is atypical, many patients present with infertility, which may not be accompanied by TB. In addition, general hospitals lack a specific understanding of TB. Before performing IVF-ET, no routine screening was conducted for FGTB as the cause of infertility, leading to direct IVF-ET in patients with FGTB and eventually resulting in pregnancy with TB, causing serious damage to patients who were infertile [16]. Both IVF-ET and natural conception combined with TB have been reported [17–19]; however, case-controlled studies of the two modes of conception with TB are relatively rare.

In the two groups in the current study, the average age of the patients in the IVF-ET conception group was higher than that of those in the natural conception group. Advanced age is a high-risk factor for gestational diabetes [20], which is related to the occurrence and recurrence of TB [21]. Further stratification of patient age and blood sugar was not performed in this study, and additional studies are thus needed.

In this study, 4 patients (30.77%) in the IVF-ET conception group tested positive for *M. tuberculosis*, and 16 patients (43.24%) in the natural conception group tested positive for this bacterium. The positive rate of *M. tuberculosis* detection in this study is consistent with a previous study [22]. The special endocrine and physiological status of pregnancy is closely related to the occurrence of TB. During pregnancy, autonomic dysregulation, endocrine and metabolic disorders and increased ovarian hormones put the lungs in a state of congestion. Meanwhile, the maternal thyroid hormone, adrenal corticoid secretion, basal metabolic rate and energy consumption also increase, resulting in a rise in pulmonary capillary permeability. At this point, tubercle bacilli are more likely to grow and multiply in the lungs, leading to TB and extrapulmonary TB [18]. In the clinical and imaging manifestations, the patients in the IVF-ET conception group were more likely to develop fever than those in the natural conception group. The count of decidual natural killer cells (dNK) cells and the secretion of some intracellular cytokines in dNK and dγδT cells decreased to an extent in the women with an IVF-ET pregnancy, suggesting that the immune microenvironment at the maternal foetal interface may have changed [23]. The patients in the IVF-ET group were more likely to be infected with haematogenous disseminated TB and extrapulmonary TB. Intracranial TB in the IVF-ET group was associated with more severe disease, longer hospital stays and a higher proportion of severe cases. The occurrence of pregnancy with TB may be related to the inhibitory effect that increased oestrogen and progesterone have on T lymphocytes in women during pregnancy [24]. Studies have confirmed that the peak value of oestrogen in the serum of patients with IVF-ET conception is several or even dozens of times higher than in patients with natural conception [25]. In ovulation induction, oestrogen and progesterone suddenly elevate in patients with IVF-ET conception, which is conducive to the growth and reproduction of *M. tuberculosis* and may be an exacerbating factor in the disease’s progression. In the imaging manifestations of pregnancy complicated with TB in this study, in addition to the haematogenous disseminated TB, the morphology of secondary TB was different from that in the general population. The proportion of pregnancy combined with a secondary pulmonary TB cavity and the tree-in-bud sign was higher, which may be related to the special physiological and immunological status of pregnancy [26]. Some researchers believe that a relationship exists between a secondary pulmonary TB cavity and a positive smear [27], which may explain why more than 40% of the patients tested positive for the *M. tuberculosis* aetiology in the natural conception group. Accordingly, the relationship between a pulmonary TB cavity and a positive smear has important implications for disease control and prevention.

The treatment of pregnancy with TB is similar to that in non-pregnancy and should be early, combined, moderate and regular and should follow a complete process [28]. The specific treatment plan depends on the gestational week and the progression of the disease. Once diagnosed, the patient should immediately undergo anti-TB treatment. Streptomycin should not be used, as it can cause foetal cranial nerve damage. In addition, relevant adjuvant therapy should be provided. Regarding pregnancy outcomes, the IVF-ET conception group had a higher proportion of pregnancy terminations due to the mothers’ severe conditions. In this study, only 2 patients continued pregnancy until birth; the condition of 1 of the infants deteriorated after natural delivery at 28 weeks, and the newborn died following abandonment of the treatment. In the natural conception group, 51.35% of the patients chose to continue their pregnancy after receiving anti-TB treatment. However, further follow-ups are needed to observe whether anti-TB treatment affects post-natal growth and development.

The limitation of this study is that it was a single-centre study with a relatively small sample size.

In conclusion, the pregnancy of patients with IVF-ET is affected more strongly by TB than natural pregnancies. More attention must be paid to TB screening in patients undergoing IVF-ET pregnancy.

### Electronic supplementary material

Below is the link to the electronic supplementary material.

Supplementary Material 1

## Data Availability

The datasets used or analyzed during the current study are available from the corresponding author on reasonable request.
